# Association between free T3 and sarcopenia in euthyroid older patients with Hashimoto’s thyroiditis

**DOI:** 10.3389/fendo.2025.1603560

**Published:** 2025-08-15

**Authors:** Jin-Liang Chen, Yuan Gao, Qian Xiao

**Affiliations:** ^1^ Department of Endocrinology and Metabolism, The Second Affiliated Hospital, Zhejiang University School of Medicine, Hangzhou, China; ^2^ Department of Geriatrics, the First Affiliated Hospital of Chongqing Medical University, Chongqing, China

**Keywords:** thyroid hormones, FT3, sarcopenia, Hashimoto’s thyroiditis, elderly

## Abstract

**Purpose:**

Growing evidence suggests that thyroid hormones play an important role in the process of sarcopenia during aging. The aim of our study was to investigate whether thyroid hormones have an association with age-related sarcopenia in euthyroid geriatric patients with Hashimoto’s thyroiditis (HT).

**Methods:**

A total of 442 euthyroid older patients with Hashimoto’s thyroiditis were included in this cross-sectional study. Sarcopenia was diagnosed according to the Asian Working Group for Sarcopenia (AWGS) 2019 criteria. Body composition, grip strength and gait speed were assessed in participants. Concentrations of thyroid hormones were determined by immunoassays. Logistic regression analyses were carried out to assess the association between free T3 (FT3) levels and sarcopenia risk.

**Results:**

Compared to non-sarcopenic patients, FT3 levels were found to be lower in the sarcopenic group (2.92 pg/ml vs 3.00 pg/ml, p<0.05). Multiple linear regression analysis showed a significant positive association between FT3 and hand grip strength, gait speed, skeletal muscle mass. Multivariable logistic regression analysis showed that FT3 levels were independently associated with sarcopenia (odds ratio 0.533 [95% confidence interval 0.343, 0.829], p=0.005) and low gait speed, low hand grip strength, low skeletal muscle index.

**Conclusion:**

Higher FT3 levels within normal range was positively associated with skeletal muscle mass, hand grip strength and physical function in elderly euthyroid individuals with HT.

## 1 Introduction

Sarcopenia, defined as the age-related loss of skeletal muscle mass and function, has emerged as a significant health concern among the elderly population. It has been one of the most common geriatric syndromes that is associated with multiple adverse health outcomes, such as frailty, falls, hospitalization and increased mortality (1). Since 2016, sarcopenia has been recognized as an independent disease with an International Classification of Disease-10 (ICD-10) Code (M62.84) (2). However, the detailed mechanisms underlying sarcopenia remain unclear. Multiple factors such as inflammation, malnutrition, immobility, and changes in hormone levels contribute to sarcopenia (3).

Thyroid hormones, namely triiodothyronine (T3) and thyroxine (T4), produced and released by the thyroid gland, have a profound impact on various tissues and organs, including skeletal muscle. Thyroid hormones play a significant role in skeletal muscle development, metabolism, and function. They contribute to muscle growth, regulate metabolic processes, influence protein turnover, and impact muscle strength (4). Thyroid dysfunction is one of the most common endocrine abnormalities in older individuals (5), whether hypothyroidism (low thyroid hormone levels) or hyperthyroidism (excess thyroid hormone levels), can have detrimental effects on skeletal muscle, both hypothyroidism and hyperthyroidism can lead to muscle weakness and atrophy (6, 7).

Hashimoto’s thyroiditis (HT) is an autoimmune disorder characterized by chronic inflammation of the thyroid gland, ultimately leading to subclinical hypothyroidism or overt hypothyroidism. It is one of the most prevalent thyroid diseases in older individuals (8).

It has been proved that hypothyroidism leads to muscle atrophy and weakness. However, it is still unclear that whether thyroid hormones are correlated with skeletal muscle mass and function in subclinical hypothyroidism or euthyroid subjects. Recent studies have investigated the correlation between subclinical hypothyroidism and sarcopenia or the sarcopenia components in older people, however the results still remained controversial (9, 10). Other studies have discussed the association of thyroid hormone concentrations with sarcopenia in euthyroid elderly subjects (11–13), and a systematic review revealed that low levels within the normal range of free triiodothyronine (FT3), high levels within the normal range of free thyroxine (FT4), and lower thyroid hormone ratio (FT3/FT4) may contribute to a reduced muscle function, which seems more evident in older males (14).

Previous studies have demonstrated a potential link between thyroid hormones and sarcopenia in various populations (10, 12, 15, 16). However, few studies have specifically focused on the association between thyroid hormones and sarcopenia in euthyroid older patients with HT. Our study specifically targets euthyroid older adults with HT, a group characterized by chronic autoimmune inflammation. This is critical because HT is the most common cause of hypothyroidism and is prevalent in aging populations (8). Despite being euthyroid, these patients may exhibit subtle thyroid dysfunction or inflammation that could accelerate sarcopenia, a hypothesis underexplored in prior research.

We hypothesized that lower thyroid hormone levels within the normal range are associated with reduced muscle mass, strength, and physical performance in euthyroid older adults with HT.

## 2 Materials and methods

### 2.1 Study design and participants

This is a cross-sectional study utilizing data from the prospectively maintained Frailty and Sarcopenia Specialist Clinic database at the Geriatric center of The First Affiliated Hospital of Chongqing Medical University. The study population comprised all consecutive participants aged ≥60 years who underwent a standardized comprehensive assessment for sarcopenia and had thyroid function tests (including thyroid antibodies) performed during their initial visit to the Frailty and Sarcopenia Specialist Clinic between August 1, 2014, and August 1, 2021. This specialist clinic is dedicated to the evaluation and management of frailty and sarcopenia in older adults. Patients were consecutively identified and screened for eligibility from the clinic database during the recruitment period. To be included in the final analysis, patients had to meet the following criteria: (1) completed the full sarcopenia assessment protocol per AWGS 2019 guidelines (including BIA for muscle mass, handgrip strength, and gait speed measurement); (2) completed serum thyroid function tests (TSH, FT3, FT4, TT3 and TT4), thyroid peroxidase antibody (TPOAb) and thyroglobulin antibody (TGAb) results; (3) underwent thyroid ultrasound examination during the initial clinic visit; (4) met the diagnostic criteria for HT while euthyroid: elevated TPOAb (>9 IU/mL) with or without elevated TGAb (>4 IU/mL) and characteristic HT findings on ultrasound (decreased echogenicity, heterogeneity, hypervascularity, hypoechoic micronodules) (8). Participants were excluded from the study based on any of the following: (1) disabled older adults; (2) a history of thyroid surgery, or iodine-131 ablation therapy, current use of levothyroxine or other thyroid hormone replacement therapy, other medication use that has the potential to disrupt thyroid function; (3) abnormal thyroid hormone levels; (4) any malignant tumors; (5) oral glucocorticoids or immunosuppressive drugs; (6) severe renal dysfunction (eGFR<30 mL/min/1.73 m^2^); (7) trans-aminase abnormality (ALT, ASL>3-fold the upper limit of normal range); (8) severe heart failure (New York Heart Association Class III- IV); (9) acute exacerbation of chronic obstructive pulmonary disease; (10) acute stroke or myocardial infarction; (11) other diseases may affect muscle metabolism (e.g. inflammatory myopathy, Parkinson’s disease, et al). After applying inclusion and exclusion criteria, a total of 442 eligible patients were included in the final analysis (204 diagnosed with sarcopenia and 238 without sarcopenia). This study was approved by the ethical Committee of the First Affiliated Hospital of Chongqing Medical University (K2023-085), and all participants gave written informed consent.

### 2.2 Sarcopenia assessments

The diagnostic criteria of sarcopenia is based on the Asian Working Group for Sarcopenia (AWGS) 2019 (17). According to the AWGS 2019 consensus, sarcopenia is still described as age-related loss of skeletal muscle mass plus low skeletal muscle strength and/or low physical performance.

Body composition was measured by Bioelectrical Impedance Analysis (BIA) (InBody S10; InBody, Seoul, Korea). Skeletal muscle index (SMI) (kg/m^2^) was calculated by normalizing appendicular skeletal muscle mass (ASM) to height in meters squared. As recommended by AWGS 2019, the cutoff thresholds for low muscle mass in sarcopenia as <7.0 kg/m^2^ in men and <5.7 kg/m^2^ in women by BIA.

Muscle strength was determined by hand grip strength measurement. The dominant hand was measured with a Jamar dynamometer in a sitting position with elbow in 90° flexion and wrist in neutral position. The maximum reading of at least 2 trials was recorded as hand grip strength and the results were expressed in kilograms (kg). According to AWGS 2019, handgrip <28.0 kg for men and <18.0 kg for women were regarded as low muscle strength.

Gait speed was measured by a trained nurse using a standardized protocol: Participants walked a 6-meter distance at their usual pace, and the time was recorded with a stopwatch. The gait speed (m/s) was then calculated. AWGS 2019 recommends gait speed <1.0 m/s as low gait speed.

### 2.3 Thyroid hormones, other laboratory and clinical measurements

Total triiodothyronine (TT3), total thyroxine (TT4), FT3, FT4 and TSH were determined with electro-chemiluminescence method (Unicel DXI 800 Immunoassay were: TSH: 0.01-0.02 μIU/mL. The manufacturer-defined reference intervals were: TT3: 0.66-1.61 ng/ml, TT4: 5.44-11.85 μg/dl, FT3: 2.01-4.82 pg/ml, FT4: 0.59-1.25 ng/dl, TSH: 0.56-5.91 μIU/ml, TPOAb: 0-9 IU/ml, TGAb: 0-4 IU/ml. Hormone levels within ±10% of reference limits underwent duplicate retesting; final values were averaged for classification. Hemoglobin levels were measured using a Mindray BC-6800 automated hematology analyzer (Mindray, China). Serum albumin, ALT, AST, blood urea nitrogen, creatinine and uric acid were measured using an automatic biochemical analyzer (Modular DDP, Roche). Serum lipids were measured enzymatically by an automatic analyzer (Model 7080; Hitachi, Tokyo, Japan) with reagents purchased from Leadman Biochemistry Co. Ltd. (Beijing, China). The blood samples of all subjects were obtained after fasting for 8 hours, and then sent to the clinical laboratory in hospital.

The information regarding demographics, medical history, alcohol and tobacco history was collected through experienced physicians. Smokers were operationally defined as individuals who reported having consumed a cumulative lifetime total of at least 100 cigarettes (or equivalent tobacco products) and confirmed active tobacco use within the preceding 28 days based on self-reported data (18). According to the National Institute on Alcohol Abuse and Alcoholism, drinkers were defined as participants who self-reported consuming ≥12 alcoholic drinks cumulatively in their lifetime and any alcohol intake within the preceding 12 months. All the patients underwent physical examination including height, weight and blood pressure, body mass index (BMI) was calculated by dividing the person’s weight (kg) by the square of height (m^2^).

### 2.4 Statistical analysis

Continuous variables were tested for normality using Shapiro-Wilk tests and Q-Q plots. Normally distributed continuous variables were presented as mean ± SD and tested by t-test. Non-normally distributed continuous variables were presented as the medians (interquartile ranges, IQRs) and tested by Mann-Whitney U-tests. Categorical variables were presented as percentage and tested by Chi-square test. Multiple linear regression analysis was performed to assess the association between FT3 (independent variables) and hand grip strength, gait speed, SMI, fat free mass (FFM), upper limbs muscle mass (ULMM) and lower limbs muscle mass (LLMM) (dependent variables) adjusted for several models (Model 1 was unadjusted; Model 2 adjusted for age, sex, alcohol and smoking; Model 3 adjusted for model 2 + diabetes, high blood pressure, coronary heart disease and hemoglobin), respectively. Univariate and multivariate logistic regression were performed to calculate the odds ratio (OR) and 95% confidence interval (CI), and to evaluate the risk of FT3 on sarcopenia. All data were analyzed using SPSS 22.0, and a p value <0.05 was regarded as statistically significant.

## 3 Results

### 3.1 Basic characteristics of study population

**Figure 1:**
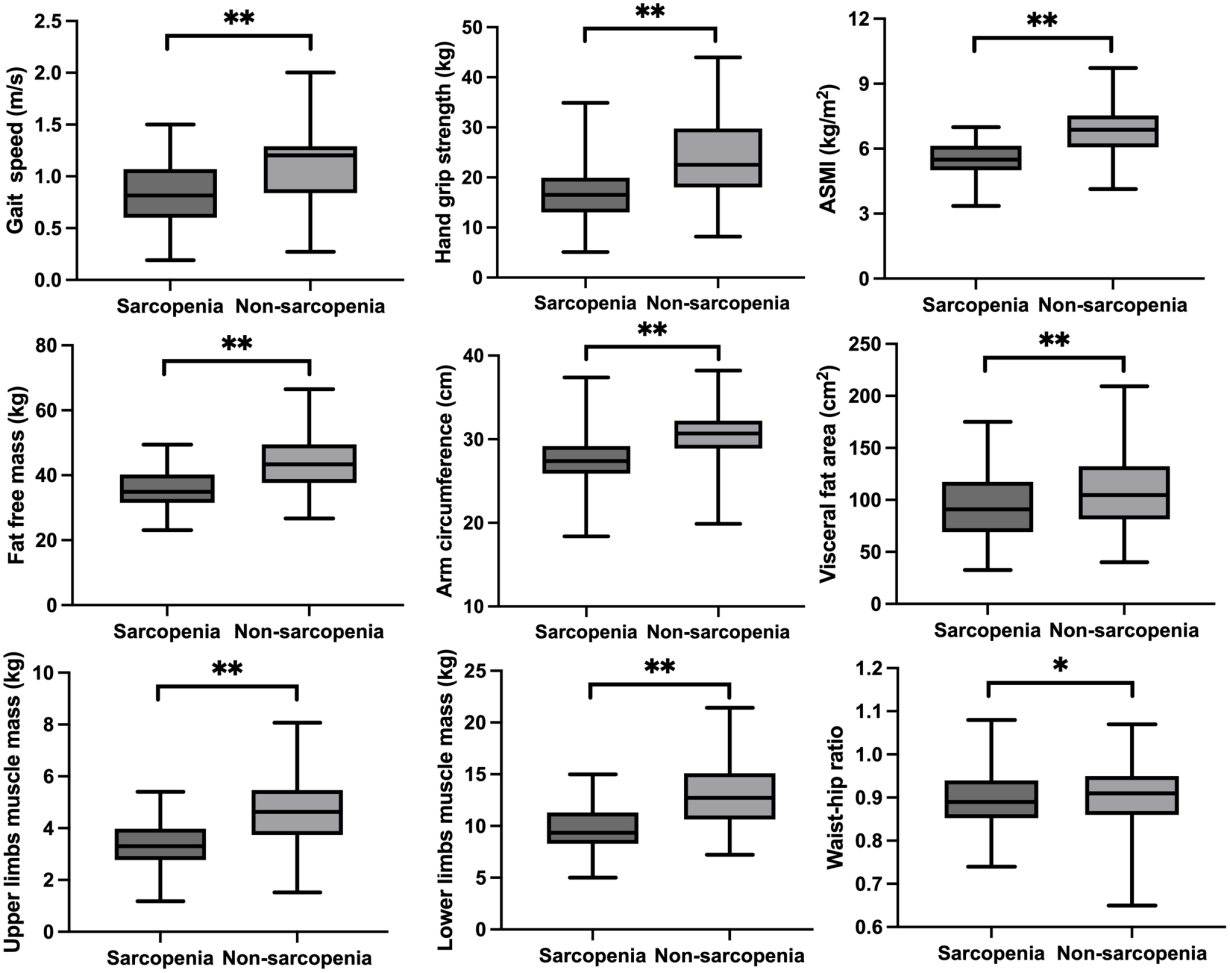
The sarcopenia components and anthropometric indicators between sarcopenia and no-sarcopenia groups. *p<0.05, **p<0.01.

Participants’ basic characteristics are presented in **Table 1**. A total of 442 participants were included in this study, involving 204 subjects with sarcopenia (mean age: 79.55 ± 7.58 years) and 238 subjects without sarcopenia (mean age: 75.56 ± 7.03 years). Compared to subjects without sarcopenia, those with sarcopenia had a lower BMI (25.63 ± 3.24 vs. 22.25 ± 3.03, p=0.001). The proportion of smoking and drinking in sarcopenia group was slightly lower than in non-sarcopenia group. There was no difference in the prevalence of diabetes, high blood pressure and coronary heart disease between the two groups. Subjects with sarcopenia exhibited lower levels of hemoglobin compared to people without sarcopenia. Regarding thyroid hormones, FT3 levels were significantly lower in sarcopenia group than that in non-sarcopenia group, and no significant differences in TT3, TT4, FT4 and TSH levels were detected between groups. Similarly, no significant differences in TPOAb and TGAb levels were observed between the two groups. As shown in **Figure 1**, we compared sarcopenia components and anthropometric indicators between the two groups. Gait speed, handgrip strength and ASMI of subjects in sarcopenia group were significantly lower than in non-sarcopenia group. With respect to anthropometric indicators, we found that fat free mass, arm circumference, visceral fat area, upper limbs muscle mass, lower limbs muscle mass and waist-hip ratio were significantly lower in sarcopenia group than those in non-sarcopenia group.

**Table 1:** Characteristics of study population with or without sarcopenia.

	Sarcopenia	Non-Sarcopenia	p value
Socio-demographics			
Male/Female (n)	82/122	98/140	0.834
Age (year)	79.55 ± 7.58	75.56 ± 7.03	0.001*
BMI (Kg/m^2^)	22.25 ± 3.03	25.63 ± 3.24	0.001*
Smoking (%)	21.9	30.2	0.049*
Alcohol (%)	16.4	23.4	0.070
Health status			
Diabetes (%)	35.3	42.9	0.105
High blood pressure (%)	68.1	74.4	0.148
Coronary heart disease (%)	49.0	44.1	0.303
Laboratory parameters			
Hemoglobin (g/L)	125 (18)	130.5 (23)	0.001*
Albumin (g/L)	40 (5)	41 (6)	0.186
ALT (U/L)	18 (13)	17 (13)	0.962
AST (U/L)	19 (8)	20 (9)	0.429
BUN (mmol/L)	5.6 (2.5)	5.65 (2.1)	0.804
Creatinine (μmol/L)	71 (20)	73 (22)	0.624
Urine acid (μmol/L)	315.5 (128)	330.5 (107)	0.233
Triglyceride (mmol/L)	1.13 (0.67)	1.20 (0.80)	0.191
Total Cholesterol (mmol/L)	4.28 (1.42)	4.22 (1.37)	0.635
HDL-cholesterol (mmol/L)	1.26 (0.51)	1.26 (0.48)	0.866
LDL-cholesterol (mmol/L)	2.49 (1.26)	2.41 (1.25)	0.589
Thyroid hormones			
TT3 (ng/ml)	0.92 (0.21)	0.94 (0.25)	0.089
TT4 (μg/dl)	7.61 (1.93)	7.49 (1.75)	0.066
FT3 (pg/ml)	2.92 (0.56)	3.00 (0.56)	0.036*
FT4 (ng/dl)	0.90 (0.20)	0.90 (0.19)	0.426
TSH (μIU/ml)	1.95 (1.43)	1.94 (1.72)	0.464
Thyroid antibodies			
TPOAb (IU/mL)	415.5 (490)	462 (511)	0.383
TGAb (IU/mL)	428.5 (484)	458 (519)	0.571

Data are presented as mean ± SD, median (IQR), or n (%).

BMI, body mass index; ALT, alanine aminotransferase; AST, aspartate aminotransferase; BUN, blood urea nitrogen; HDL, high-density lipoprotein; LDL, low-density lipoprotein; TT3, total triiodothyronine; TT4, total thyroxine; FT3, free triiodothyronine; FT4, free thyroxine; TSH, thyroid stimulating hormone; TPOAb, thyroid peroxidase antibody; TGAb, thyroglobulin antibody.

*Statistically significant difference.

### 3.2 The relationship between FT3 and measures of sarcopenia

**Figure 2:**
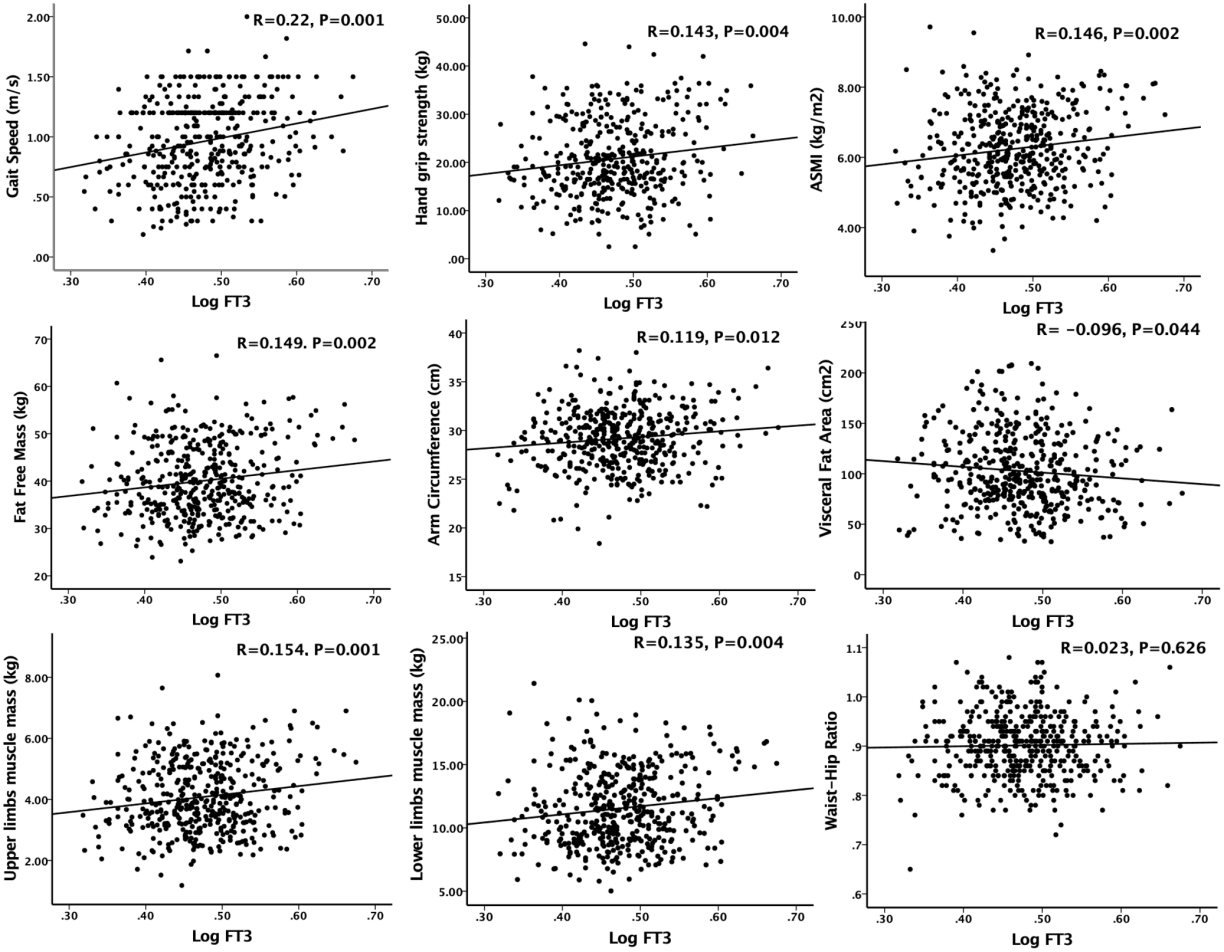
Simple correlation analysis of sarcopenia components and anthropometric indicators with serum FT3 concentration. Logarithm-transformed values of serum FT3 was used for analysis.

As shown in **Figure 2**, in the simple correlation analysis, serum FT3 had a significant positive correlation with gait speed (R=0.22, p=0.001), handgrip strength (R=0.143, p=0.004) and ASMI (R=0.146, p=0.002). Serum FT3 also had a significant positive correlation with fat free mass (R=0.149, p=0.002), arm circumference (R=0.119, p=0.012), upper limbs muscle mass (R=0.154, p=0.001) and lower limbs muscle mass (R=0.135, p=0.004). There was a negative correlation between serum FT3 levels and visceral fat area (R=-0.096, p=0.044). However, no significant correlation was observed between serum FT3 and waist-hip ratio (R=-0.023, p=0.626). In the multiple linear regression analysis, as shown in **Table 2**, model 1 was unadjusted; Model 2 adjusted for age, sex, alcohol and smoking (VIF<3); Model 3 adjusted for model 2 + diabetes, high blood pressure, coronary heart disease and hemoglobin (VIF<3). Serum FT3 had a significant positive association with handgrip strength, gait speed, SMI, fat free mass, upper limbs muscle mass and lower limbs muscle mass for model 1, model 2 and model 3 (p<0.05).

**Table 2:** Multiple linear regression analysis of serum FT3 concentration with measures of sarcopenia.

FT3	Model 1		Model 2		Model 3	
	Standard β	p value	Standard β	p value	Standard β	p value
HGS	0.151	0.003**	0.125	0.018*	0.090	0.042*
Gait speed	0.218	<0.001**	0.213	<0.001**	0.163	0.002**
SMI	0.155	0.001**	0.088	0.015*	0.077	0.041*
FFM	0.159	0.001**	0.075	0.025*	0.071	0.040*
ULMM	0.162	0.001**	0.078	0.022*	0.063	0.048*
LLMM	0.147	0.002**	0.067	0.045*	0.065	0.048*

Model 1: unadjusted. Model 2: adjusted for age, sex, alcohol and smoking. Model 3: Model 2+adjusted for diabetes, high blood pressure, coronary heart disease and hemoglobin.

HGS, hand grip strength; SMI, skeletal muscle index; FFM, fat free mass; ULMM, upper limbs muscle mass; LLMM, lower limbs muscle mass.

*p<0.05, **p<0.01.

### 3.3 The effect of FT3 on sarcopenia and sarcopenia components

As shown in **Table 3**, univariate and multivariable-adjusted models were used for evaluating the relationship between FT3 and sarcopenia. In the univariate logistic analysis, increased serum FT3 levels significantly decreased the risk of sarcopenia (OR 0.533 [95%CI 0.343, 0.829], p=0.005). After adjusting for Model 2 (age, sex, alcohol and smoking), the relationship between FT3 and sarcopenia remained the same (OR 0.560 [95%CI 0.356, 0.883], p=0.012). After adjusting for Model 3 (Model 2+diabetes, high blood pressure, coronary heart disease and hemoglobin), the result revealed that FT3 still had a significant negative correlation with the risk of sarcopenia (OR 0.597 [95%CI 0.369, 0.966], p=0.036). The results showed that FT3 levels decreased the risk of sarcopenia in elderly individuals with HT.

**Table 3:** Univariate and multivariable analysis for logistic regression of sarcopenia.

	Univariate		Multivariate (Model 2)		Multivariate (Model 3)	
	OR (95% CI)	P value	OR (95% CI)	P value	OR (95% CI)	P value
FT3	0.533 (0.343, 0.829)	0.005**	0.560 (0.356, 0.883)	0.012*	0.597 (0.369, 0.966)	0.036*

Univariate: univariate logistic regression analyses for the association between FT3 and sarcopenia. Multivariate: multivariate logistic regression analyses to test whether FT3 are independently associated with sarcopenia. Model 2: Adjusted for age, sex, alcohol and smoking. Model 3: Adjusted for Model 2+diabetes, high blood pressure, coronary heart disease and hemoglobin.

FT3, free triiodothyronine; OR, odds ratio; 95% CI, 95% confidential interval.

*p<0.05, **p<0.01.

We further discussed the potential risk factor between FT3 and sarcopenia components. As shown in **Table 4**, univariate and multivariable-adjusted models were used for evaluating the relationship between FT3 and sarcopenia components. The unadjusted model showed the level of FT3 reduced the risk of low gait speed, low hand grip strength and low SMI. After multivariable adjustment for the potential explanatory factors for sarcopenia (model 2 and model 3), the results were consistent with the unadjusted model.

**Table 4:** Univariate and multivariable analysis for logistic regression of low gait speed, strength and skeletal muscle mass.

	Univariate		Multivariate (Model 2)		Multivariate (Model 3)	
	OR (95% CI)	P value	OR (95% CI)	P value	OR (95% CI)	P value
Low gait speed	0.524 (0.333, 0.824)	0.005**	0.508 (0.350, 0.899)	0.009**	0.551 (0.344, 0.884)	0.013*
Low hand grip strength	0.611 (0.387, 0.963)	0.034*	0.603 (0.378, 0.964)	0.034*	0.597 (0.371, 0.963)	0.036*
Low SMI	0.640 (0.417, 0.982)	0.041*	0.659 (0.424, 0.998)	0.049*	0.667 (0.416, 1.067)	0.091

Univariate: univariate logistic regression analyses for the association between FT3 and low gait speed, low hand grip strength and low SMI. Multivariate: multivariate logistic regression analyses to test whether FT3 are independently associated with gait speed, strength and SMI. Model 2: Adjusted for age, sex, alcohol and smoking. Model 3: Adjusted for model 2+diabetes, high blood pressure, coronary heart disease and hemoglobin.

SMI, skeletal muscle index; FT3, free triiodothyronine; OR, odds ratio; 95% CI, 95% confidential interval.

*p<0.05, **p<0.01.

## 4 Discussion

The findings of this study demonstrated that FT3, rather than FT4, TT4, TT3 and TSH, was positively associated with skeletal muscle mass (FFM, ULMM, LLMM), muscle strength and physical function among Chinese euthyroid elderly subjects with HT. The results suggest that thyroid hormone status, specifically FT3, may play a crucial role in the development and progression of sarcopenia in HT patient population. Consistent with previous studies, FT3 is associated with the development of sarcopenia or the components of sarcopenia (12, 13, 19). These results might suggest a possible correlation that lower FT3 levels are associated with a higher prevalence and severity of sarcopenia.

Consistent with most published studies, our results suggested that high-normal FT3 levels were correlated with good muscle mass, muscle strength and physical performance. However, Chen J et al. found that TT3, rather than FT3, was a more stable and practical indicator to evaluate the relationship between sarcopenia and thyroid hormone in the elderly euthyroid population (20), one of the reasons for this specificity is that the subjects in their study were older than the others, most of whom were the old-old (more than 80 years-old), for FT3 is easily influenced by age, season and temperature (21, 22).

The underlying mechanisms linking FT3 levels and sarcopenia in euthyroid individuals with HT require further exploration. One possible explanation is the impact of reduced thyroid hormone activity on muscle metabolism and protein synthesis. Thyroid hormones, including FT3, are known to regulate basal metabolic rate and protein turnover, which are essential for muscle maintenance and growth (23, 24). Decreased FT3 levels may lead to a state of reduced anabolism, resulting in muscle wasting and the development of sarcopenia. Another possible mechanism could involve the influence of chronic inflammation on both thyroid function and skeletal muscle. HT is characterized by autoimmune-mediated inflammation of the thyroid gland. This chronic inflammatory state, in turn, may contribute to systemic inflammation, which has been linked to muscle wasting and dysfunction (25).

HT increased the risk of hypothyroidism or subclinical hypothyroidism. Overt hypothyroidism is proved to be associated with poor gait speed and falls, and a clear indicator for thyroid hormone treatment (26). Subclinical hypothyroidism affects up to 10% of the adult population, and it is more prevalent in the elderly individuals (27), recent RCT studies revealed that hormone replacement therapy of subclinical hypothyroidism did not affect muscle mass, strength and function in individuals over 65 years old (28). To date, no evidence showed that whether hormone treatment in euthyroid individuals to increase FT3 levels will benefit the skeletal muscle.

Monitoring FT3 levels in euthyroid individuals with HT may serve as a useful tool for identifying individuals at higher risk of developing sarcopenia. Early detection and intervention in this population could help prevent or mitigate the progression of sarcopenia and its associated adverse outcomes, such as falls, fractures, and loss of functional independence. Furthermore, optimizing thyroid hormone replacement therapy, specifically targeting FT3 levels, may be a potential therapeutic strategy to attenuate sarcopenia in euthyroid individuals with HT.

Interestingly, despite the autoimmune nature of Hashimoto’s thyroiditis, our study found no significant differences in TPOAb or TGAb levels between sarcopenic and non-sarcopenic groups. This may be attributed to several factors: (1) TPOAb and TGAb are specifically directed against thyroid antigens and likely lack a direct pathogenic role in skeletal muscle tissue (29). Their levels primarily reflect thyroid-specific autoimmunity rather than processes driving extra-thyroidal sarcopenia. (2) Thyroid autoantibody levels often peak early in the HT disease course and may plateau, while sarcopenia develops slowly over time in the context of aging and the long-term hormonal milieu (30). Thus, a single-point measurement of antibodies in euthyroid individuals might not capture the relevant timeframe for muscle impact, whereas FT3 levels directly reflect current bioavailable hormone influencing muscle anabolism. (3) Although antibodies are useful diagnostic markers for HT, the development of sarcopenia in euthyroid patients may involve other immune dysregulation or inflammatory pathways, either independent of or downstream to these specific thyroid autoantibodies. Therefore, while marking the underlying autoimmune thyroiditis, TPOAb/TGAb levels themselves do not appear to predict sarcopenia risk in this stable, euthyroid geriatric HT population, underscoring the more direct importance of maintaining optimal FT3 levels for muscle health.

Several limitations should be acknowledged in our study. First, although we adjusted for key potential confounders in our multivariate models, residual confounding from unmeasured factors (e.g., physical activity levels, nutritional status) may still exist. Second, the cross-sectional design limits our ability to establish causality and determine the temporal relationship between FT3 levels and sarcopenia. Longitudinal studies are warranted to elucidate the causal relationship of the observed association. Our findings are not generalizable to patients on thyroid hormone replacement, as they were excluded from this study. Additionally, potential biases might exist due to a small sample size, and the findings might not be applicable to other populations due to the inclusion of individuals with HT.

In conclusion, our study demonstrated that FT3 levels were positively correlated with skeletal muscle mass, hand grip strength, arm circumference and physical activity in elderly euthyroid subjects with HT. We hypothesized that maintaining higher FT3 concentrations within the normal range is crucial for preserving muscle mass and function in elderly individuals. However, further studies are needed to validate this hypothesis.

## Data Availability

The raw data supporting the conclusions of this article will be made available by the authors, without undue reservation.
